# Our Bureau of Information

**Published:** 1912-03-09

**Authors:** 


### OUR BUREAU OF INFORMATION.

### The Needs of Epileptics.

Epileptics are a class of sufferers who deserve a special amount of sympathy because of the periodicity—often irregular—of their attacks. Perhaps this condition helps in the development of the insanity and imbecility in which epilepsy sometimes ends. It is only comparatively lately that the needs of sufferers from this disease have been realised, and it is doubtful even yet if the supply of suitable institutions is adequate. London is far ahead of the rest of the country, for not only has it three hospitals for the paralysed and epileptic, but there is a County Epileptic Colony at Ewell, where the average number of inmates is 355, and two colonies which are partly self-supporting and partly maintained by private charity —one at Chalfont St. Giles, which accommodates 263 inmates—men, women and children—and another for women and children only at Godalming, where ninety-eight women and girls are received. In these two, payment varying from 10s. to 14s. a week for each patient is asked, the remainder of the necessary cost of maintenance being obtained from outside sources. In these colonies the inmates are employed at work suitable to their strength and capacity, and do something towards earning their own living.

But what of the rest of the country? There is a home for epileptics at Liverpool, where the fees charged run from half a guinea to two guineas a week. The inmates of this home number nearly 300, and a special feature is the education, training, and maintenance of seventy children in a special house, certified by the Board of Education. There is also a home near Manchester—at Alderley Edge, where there are five houses each capable of containing twenty-four patients. Of these three are for men and two for women, and a uniform charge of 14s. a week is made. Greater privacy can be obtained in two other houses for patients who can afford to pay for it. A single-bedded room and private sitting-room can be had for £2 10s. a week. But in Scotland there is only one colony for epileptics—at Bridge of Weir. Here there are sixty beds available, and the charge is 10s. a week. But Ireland is in even worse case. There one finds no such colony as exists on this side of the Channel, and only one special hospital—the Belfast Hospital for Diseases of the Nervous System, Paralysis, and Epilepsy—which contains but twenty beds! Neither in Scotland nor in Ireland do there appear to be any public institutions like the colony at Ewell, where the poorest epileptics can be received, and we fear that when these unfortunates are not kept at home they are placed in lunatic asylums, a course of action which is calculated to affect their minds most injuriously.

Here, then, is a work for both private beneficence and public duty. In the newspaper one often reads of deaths through falling into the fire or being strangled through the pressure of ordinary clothes in cases where the victim is "subject to fits." These are the cases for which homes, either free or making but a small charge, are needed.

### Needs and Workers.

### Home for Hospital Out-patients.

"Outsider" writes: I came to London to see a doctor at a hospital, and he wishes me to remain in town for some weeks, receiving out-patient treatment at the hospital. Can you recommend a suitable place for me to stay at? I cannot afford London lodgings.

[The House of Charity, 1 Greek Street, Soho, should meet your case. It was originally intended to provide a comfortable home for women seeking employment, but hospital out-patients are also received there. The home is under the care of the Clewer Sisters.]

### Home for a Jewish Incurable.

"Naomi" writes: I wish to get a poor woman into a hospital for incurables. The difficulty is that she is a strict Jew, and I fear that the question of food would be a difficulty in any ordinary institution.

[There is a home for Jewish incurables in the High Road, Tottenham, where your protégée's prejudices would be respected. The Secretary is Mr. L. J. Salomons.]

### Employment Wanted.

"Ferndale" writes: A young lady of twenty-one wishes to apply for a position as nurse-probationer at a hospital or public institution. Will you inform me: (1) The usual method for making such an application; (2) what references are required, and whether the names of patrons, subscribers, or officials should be included in the references in question?

[Only a children's hospital will take a girl of twenty-one as probationer. In a general hospital the usual minimum age is twenty-three. Poor-Law infirmaries, however, some of which give a good general training, take probationers at an earlier age. The candidate should write to the matron of the hospital she wishes to enter, who will send her a form of application to fill up. Allusions to "patrons, subscribers, or officials" will not help her. All information on the subject can be obtained from "How to Become a Nurse," by Sir H. Burdett, published by the Scientific Press, Southampton Street, Strand.]

### Lady Dispensers in the Colonies.

"Dersith" writes: Can you inform me whether there is a demand for lady dispensers—*i.e.* those possessing the diploma of the Apothecaries' Hall—in New South Wales?

[It is impossible for anyone on this side of the world to say definitely what chances there are for employment on the other; but the diploma of the Apothecaries' Hall will carry due weight in any portion of the British Empire. Our correspondent should look up the list of hospitals in New South Wales given in Burdett's "Hospitals and Charities," and on her arrival in Australia write to these institutions, stating her qualifications, and asking if they have any vacancies. She might also call upon dispensing chemists in Sydney, or wherever she settles, offering her services. In the Colonies there is less disposition to refuse a woman's services than is the case in the old country.]

### Rules for Correspondents.

Everyone who uses or wishes to help our Bureau of Information must be kind enough to observe the following rules:—

(1) Postal replies cannot be given. Exception will only be made in very special cases at the Editor's discretion.

(2) Queries or answers relating to different cases must be written, or preferably typed, on separate sheets of paper, and the writing must never be crossed.

(3) Questions and replies received not later than Wednesday will, as far as possible, be dealt with in our issue of the following Saturday week.

(4) Letters must have attached the name and address of the sender, with a pseudonym for publication if preferred.

(5) Applications referring to non-dependents where the need relates to business or employment must be accompanied by a postal order for 5s., when the requirement shall have publicity in these columns.

(6) All applicants for insertion in our list of Approved Homes must send a registration fee of five shillings. The omission of this leads to delay and gives avoidable trouble.

(7) *All communications* for this department *must be accompanied by the coupon* to be found at the bottom of the third page of the cover on the inside, and should be addressed to the Editor of the Bureau of Information, c/o The Hospital, 29 Southampton Street, Strand, London, W.C.

